# Fluorinated Boron-Based Anions for Higher Voltage Li Metal Battery Electrolytes

**DOI:** 10.3390/nano11092391

**Published:** 2021-09-14

**Authors:** Jonathan Clarke-Hannaford, Michael Breedon, Thomas Rüther, Michelle J. S. Spencer

**Affiliations:** 1 School of Science, RMIT University, GPO Box 2476, Melbourne, VIC 3001, Australia; jonathan.clarke-hannaford@student.rmit.edu.au; 2 CSIRO Manufacturing, Private Bag 10, Clayton South, VIC 3169, Australia; 3 CSIRO Energy, Private Bag 10, Clayton South, VIC 3169, Australia; thomas.ruether@csiro.au; 4 ARC Centre of Excellence in Future Low-Energy Electronics Technologies (FLEET), School of Science, RMIT University, GPO Box 2476, Melbourne, VIC 3001, Australia

**Keywords:** lithium metal anode, borate anion, electrolyte, SEI layer, DFT, Li-salt, battery

## Abstract

Lithium metal batteries (LMBs) require an electrolyte with high ionic conductivity as well as high thermal and electrochemical stability that can maintain a stable solid electrolyte interphase (SEI) layer on the lithium metal anode surface. The borate anions tetrakis(trifluoromethyl)borate ([B(CF_3_)_4_]^−^), pentafluoroethyltrifluoroborate ([(C_2_F_5_)BF_3_]^−^), and pentafluoroethyldifluorocyanoborate ([(C_2_F_5_)BF_2_(CN)]^−^) have shown excellent physicochemical properties and electrochemical stability windows; however, the suitability of these anions as high-voltage LMB electrolytes components that can stabilise the Li anode is yet to be determined. In this work, density functional theory calculations show high reductive stability limits and low anion–cation interaction strengths for Li[B(CF_3_)_4_], Li[(C_2_F_5_)BF_3_], and Li[(C_2_F_5_)BF_2_(CN)] that surpass popular sulfonamide salts. Specifically, Li[B(CF_3_)_4_] has a calculated oxidative stability limit of 7.12 V vs. Li^+^/Li^0^ which is significantly higher than the other borate and sulfonamide salts (≤6.41 V vs. Li^+^/Li^0^). Using ab initio molecular dynamics simulations, this study is the first to show that these borate anions can form an advantageous LiF-rich SEI layer on the Li anode at room (298 K) and elevated (358 K) temperatures. The interaction of the borate anions, particularly [B(CF_3_)_4_]^−^, with the Li^+^ and Li anode, suggests they are suitable inclusions in high-voltage LMB electrolytes that can stabilise the Li anode surface and provide enhanced ionic conductivity.

## 1. Introduction

Li metal is an ideal anode material for rechargeable Li-based batteries due to its high theoretical gravimetric capacity (3862 mAh g^−1^) that can enable much greater capacities than current state-of-the art Li-ion batteries [1]. However, significant challenges in terms of safety and practicality persist for Li metal anode-based batteries (LMBs) that originate from the thermodynamic instability of Li metal with organic solvents [2] and the long-term inefficiency of the Li plating/stripping process during cycling [3]. The repeated cycling of the Li anode can facilitate the propagation of Li dendrites and a continuous reaction between Li metal and the electrolyte, resulting in the loss of active material and a shortened cycle life [4,5,6,7,8]. One strategy that can prevent dendrite growth and prolong the cycle life of LMBs is the formation of a stable (in situ) solid electrolyte interphase (SEI) layer, which consists of a thin film of reaction products immediately formed after contact between the electrolyte and the Li anode [2,8,9,10]. Ideally, the SEI layer is compact and remains intact during cycling to prevent the loss of active materials and to minimise Li dendrite growth [4,11]. Traditional organic carbonate-based electrolytes have high volatility, flammability, and will form an SEI layer on Li metal with poor chemical stability and mechanical strength [2]. These electrolytes also possess poor stability above 4.3 V vs. Li/Li^+^ [12,13] and hence are incompatible with high-voltage cathode materials (working voltages > 4.7 V vs. Li^+^/Li^0^) such as LiNi_0.5_Mn_1.5_O_4_ that would otherwise further increase the energy densities of rechargeable batteries. Therefore, an alternative electrolyte that can stabilise the Li anode while also possessing high-voltage stability (up to 5 V vs. Li^+^/Li) is essential to ensuring the safe and reliable operation of LMBs [12,14,15,16].

Mixtures of ionic liquids (ILs) with metal salts are attractive alternative electrolytes owing to their many advantages over conventional molecular solvent-based electrolytes, such as general non-flammability and combustibility, negligible vapour pressure, high thermal and redox stability, and good conductivity [17]. It has also been shown that the anion component of ILs and Li-salts play a crucial role in determining the physicochemical and transport properties of the electrolyte and the composition of the resulting SEI layer that forms on the Li surface [18]. Yet, there are currently only a small number of anions that have good transport properties [18], have the ability to form a protective SEI layer, and possess wide electrochemical stability windows to withstand the harsh electrochemical environment of high-voltage Li batteries [19]. Previous studies have indicated that borate compounds used as Li-salts or additives can suppress Li dendrite growth through the formation of a stable fluorine- and boron-rich SEI layer [20,21,22,23]. The formation of a stable, compact, and LiF-rich SEI layer is associated with enabling the uniform deposition of Li ions through the SEI layer [24,25] and preventing corrosion of the Li anode [26], which enhances the Coulombic efficiency and cycling stability of LMBs [27,28,29,30].

The further development and design of boron-based anions has previously included a series of anions based on a central B atom and conjugated systems as ligands comprised of imidazole and pyrrole rings [31]. Density functional theory (DFT) calculations were used to assess the solubility and oxidative and reductive stability of these anions when coordinated to the Li^+^, finding an easier Li^+^ dissociation of these systems compared to lithium bis(trifluoromethanesulfonyl)imide (Li[TFSI]) and even lithium hexafluorophosphate (Li[PF_6_]), especially when multiple –CN groups are present [31]. Rüther et al. [18] also identified promising fluorine-rich borate anions. These include the homoleptic tetrakis(trifluoromethyl)borate ([B(CF_3_)_4_]^–^) and the heteroleptic pentafluoroethyltrifluoroborate ([(C_2_F_5_)BF_3_]^−^) and pentafluoroethyldifluorocyanoborate ([(C_2_F_5_)BF_2_(CN)]^−^) anions, which have all shown excellent properties when part of an IL or Li-salt [18,32,33,34,35], including decomposition temperatures above 180 °C, low melting and glass transition points, and superior electrochemical stabilities and ionic conductivities [18,32,33,34,35]. Yet, for these anions to enhance the cycling stability and longevity of high-voltage LMBs, it is critical they have high oxidative stabilities and can create a stable SEI layer on the Li metal surface [18,36] without forming unwanted gaseous by-products, with this currently unknown.

In this work, the anion–Li^+^ interaction strength, dipole moment, and charge distribution of Li[B(CF_3_)_4_], Li[(C_2_F_5_)BF_3_], and Li[(C_2_F_5_)BF_2_(CN)] were determined using density functional theory (DFT) calculations. Their stability was investigated by estimating the chemical hardness and oxidative and reductive stability limits. Furthermore, ab initio molecular dynamics (AIMD) simulations of the Li-salts on the Li(001) surface are performed to determine their chemical stability against the Li anode and the reactions leading to the formation of an SEI layer. The findings show that these anions possess a superior electrochemical stability window, ionic conductivity, and Li anode protection, making them important candidates for LMBs.

## 2. Methodology

### 2.1. Individual Anions and Li-Salts

**Figure 1:**
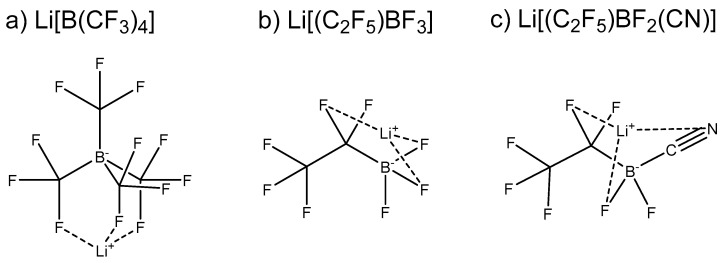
Borate anion-based Li-salts (**a**) lithium tetrakis(trifluoromethyl)borate (Li[B(CF_3_)_4_]), (**b**) lithium pentafluoroethyltrifluoroborate (Li[(C_2_F_5_)BF_3_]) and (**c**) pentafluoroethyldifluorocyanoborate (Li[(C_2_F_5_)BF_2_(CN)])investigated in this work.

All calculations involving the individual anions and Li-salts (Figure 1) were performed using the Gaussian 16 package [37] with the M06-2X [38] DFT functional and the 6-311++G(2d,p) basis set. This approach was previously determined to accurately calculate the geometry and electronic properties of similar Li-salt systems [31,39,40]. Each individual anion was optimised (in vacuum) for >20 randomly assigned coordination sites in order to locate all possible Li^+^ coordination sites (Figures S1–S4), with this process also determining the most stable geometry of each Li-salt system. All geometries reported in Figures S1–S4 were confirmed to be minima. The electronic dissociation energy (Δ*E_d_*), also commonly referred to as the binding or interaction energy, was calculated for the separation of the anion–Li^+^ into their individual components based on Equation (1):

Δ*E*_d_ = *E*_LiA_ − (*E*_A_^−^ + *E*_Li_^+^) (1)

where *E*_LiA_, *E*_A_^–^, and *E*_Li_^+^ is the total energy of the Li-salt, individual anion, and Li^+^, respectively. For the Li-salt systems, the basis set superposition error was determined to range from 3.37 to 6.99 kJ∙mol^–1^ and was incorporated in all calculations of Δ*E_d_*. Changes in the charge distribution were assessed using the natural bond orbital (NBO) method [41]. The reductive (*E*_red_) and oxidative (*E*_ox_) electrochemical stability limits were also assessed for each of the most stable Li–anion pair configurations using the previously established method in the study of Jankowski et al. [39] and summarised in the following equations:

*E*_red_ = ((Δ*G*_red_ − Δ*G*_LiA_)/*F*) + 1.46 V (2)

*E*_ox_ = ((Δ*G*_ox_ − Δ*G*_LiA_)/*F*) + 1.46 V (3)

where Δ*G*_red_, Δ*G*_ox_, and Δ*G*_LiA_ are the calculated Gibbs free energy for the relaxed Li-salt species in a reduced, oxidised, and neutral state, respectively, and *F* is the Faraday constant. The resulting potentials were adjusted by 1.46 V to align with the Li^+^/Li^0^ redox couple. Reduction and oxidation calculations were performed for the most stable Li-salt configurations with the conductor-like polarisable continuum model to implicitly solvate the Li-salts. The parameter for water was used as it was previously determined to be a suitable proxy for highly polar solvents [39].

The ionisation potential (IP) and electron affinity (EA) were calculated for each of the Li-salts. This included adiabatic (IP_A_ and EA_A_) and non-adiabatic (IP_V_ and EA_V_) variants, where the former required the Li-salts to be relaxed after removing or adding an electron. The latter is determined by a vertical reaction where there is no geometry relaxation after removing or adding electrons. The non-adiabatic (vertical) IP and EA were used to calculate the chemical hardness (η) and electronegativity (χ) using the following equations [42]:

η = (IP_v_ − EA_v_)/2 (4)

χ = (IP_v_ − EA_v_)/2. (5)

### 2.2. Li-Salt/Li(001)

The three Li-salt systems, in their most stable configurations were placed ≥ 2.5 Å above a seven-layer Li(001)-[4 × 4] surface having a ≈19 Å vacuum spacer (see [43,44] for details). AIMD simulations were performed using version 5.4.4. of the Vienna ab initio simulations package (VASP) [45,46,47,48]. A projector-augmented wave method [49] was employed, with the Perdew, Burke, and Ernzerhof [50] (PBE) exchange correlation functional and a cut-off energy of 550 eV. The atoms were relaxed so that the total energy was ≤10^−4^ eV for all systems. The effect of van der Waals forces on the ion–ion interactions was accounted for using the DFT-D3 method of Grimme et al. [51]. A 4 × 4 × 1 Γ-centred k-point mesh was used. The simulations were performed with an NVT ensemble at 298 and 358 K, with the temperature controlled by the Nosé thermostat [52]. The simulations were run for ≈6–13 ps with a time step of 0.5 fs, and the positions of the bottom two layers of the surface were kept fixed while all others were allowed to relax in all directions.

## 3. Results and Discussion

The interaction of the borate anions with the Li^+^ and Li anode are examined in two distinct parts. First, DFT calculations are used to determine the binding energy, charge distribution, dipole moment, chemical hardness, and electrochemical stability limits of the Li-salt systems. These findings are discussed relative to a series of common sulfonamide anions reported in reference [40]: [TFSI]^−^, bis(flurosulfonyl)imide ([FSI]^−^), and (fluorosulfonyl)(trifluoromethanesulfonyl)imide ([FTFSI]^−^). Subsequently, AIMD simulations are used to determine the decomposition reactions of the borate anions on the Li(001) surface at 298 K and 358 K that lead to the formation of the initial LiF-rich SEI layers.

### 3.1. DFT Calculations of Individual Anions and Li-Salts

The most stable geometries of each boron-based anion, including their charge distribution, are shown in Figures S1–S4 and Table S1, respectively. For the [B(CF_3_)_4_]^−^ and [(C_2_F_5_)BF_3_]^−^ anions, they are stable in one configuration (Figures S1a and S2a, respectively). However, for the [(C_2_F_5_)BF_2_(CN)]^−^ anion, it exists in two configurations: an *anti-* (Figure S3a) and *syn-* (Figure S4a) form, with the former being slightly more favourable by 2 kJ/mol. Hence, the *anti*-configuration is included in further analysis of its interaction with the Li^+^ and the Li(001) surface.

#### 3.1.1. Electronic Dissociation Energy, Charge Distribution, and Dipole Moment for Li-Salt Systems

The interaction strength between the Li^+^ and the anions can be assessed in terms of the electronic dissociation energy, charge distribution, and dipole moment (μ) of the Li-salt. A weaker interaction between an anion with a Li^+^ will typically feature a more positive Δ*E*_d_ value, smaller dipole moment, and high degree of charge delocalisation [18,53,54]. Such anions that interact weakly (and have extensive charge delocalisation) can lead to enhanced solubility and ionic conductivity when compared to anions that coordinate strongly to the Li^+^ [55]. Furthermore, a high degree of charge delocalisation is associated with high oxidative stability [56,57,58,59], which is a desirable feature for electrolyte components that are to be utilised in high-voltage LMBs.

The geometries of all the unique minimum energy structures of the Li-salt systems can be found in Figures S1–S4. It was shown that the addition of a Li^+^ cation to the borate-based anions almost exclusively resulted in a tri-dentate chelation configuration, which is not unexpected given the high degree of rotational freedom of the functional groups on each anion. Two configurations were determined for the symmetric [B(CF_3_)_4_]^−^ anion, consisting of the Li^+^ coordinated in a tri-dentate configuration to either three (Figure S1c) or two (Figure S1d) –CF_3_ groups, with the former being more stable. In contrast, the asymmetric [(C_2_F_5_)BF_3_]^−^ and [(C_2_F_5_)BF_2_(CN)]^−^ anions were found to have three to four times the number of Li^+^ coordination sites: six each for Li[(C_2_F_5_)BF_3_] (Figure S2) and *anti*-Li[(C_2_F_5_)BF_2_(CN)] (Figure S3), and eight for *syn*-Li[(C_2_F_5_)BF_2_(CN)] (Figure S4). The increased diversity in the Li-coordination for the asymmetric anions suggests that an enhanced dynamic coordination behaviour would occur when in solution, corresponding to an improved Li^+^ transport capability, compared to the symmetric [B(CF_3_)_4_]^−^ anion.

The analogous chemical structures of [(C_2_F_5_)BF_3_]^−^ and [(C_2_F_5_)BF_2_(CN)]^−^ result in similar Li^+^ coordination sites for both anions. The configurations where the Li^+^ is coordinated to one or more functional groups of the B atom (–F or –CN) correspond to the strongest electronic dissociation energy values for [(C_2_F_5_)BF_3_]^−^ (−577 to −546 kJ/mol) and [(C_2_F_5_)BF_2_(CN)]^−^ (−542 to −514 kJ/mol). In contrast, the tri-dentate coordination of the Li^+^ to two CF_3_ groups of [B(CF_3_)_4_]^−^ (−472 kJ/mol) or to the CF_3_CF_2_ groups of [(C_2_F_5_)BF_3_]^−^ (−487 kJ/mol) and [(C_2_F_5_)BF_2_(CN)]^−^ (−448 kJ/mol) corresponds to the weakest interaction energies for each anion. The weaker interaction calculated for these configurations of the asymmetric anions corresponds to the lower partial negative charge on the –F ligands attached to the C atoms compared to those attached directly to the B centre.

**Figure 2:**
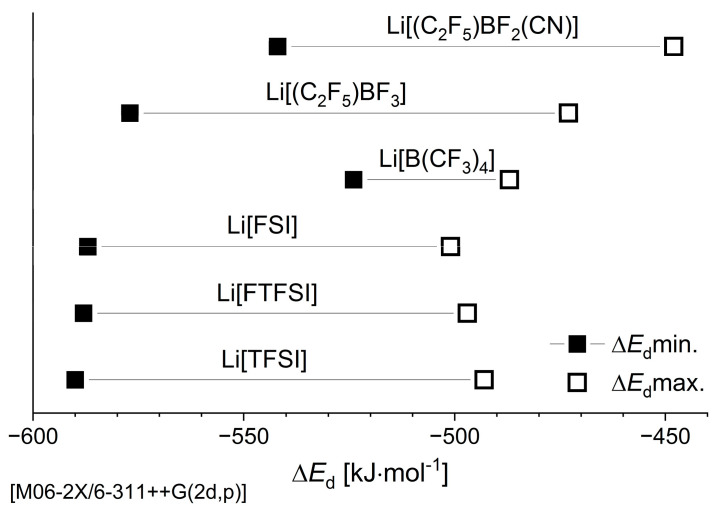
Minimum and maximum electronic dissociation energies of the three Li-salt systems from this work. The values for lithium bis(flurosulfonyl)imide Li[FSI], lithium (fluorosulfonyl)(trifluoromethanesulfonyl)imide Li[FTFSI], and lithium bis(trifluoromethanesulfonyl)imide (Li[TFSI]) are from reference [40].

The electronic dissociation energy values of the most stable (Δ*E*_d_ min.) and the least stable (Δ*E*_d_ max.) borate and sulfonamide anion-based Li-salt configurations are reported in Table S2 and visualised in Figure 2.

The order of the calculated binding energies is as follows: Li[TFSI] > Li[FTFSI] > Li[FSI] > Li[(C_2_F_5_)BF_3_] > Li[(C_2_F_5_)BF_2_(CN)] > Li[B(CF_3_)_4_]. The weaker values for the boron-based anions could facilitate easier Li^+^ dissociation compared to the sulfonamide class of anions, which is anticipated to result in higher ionic conductivities in their respective IL and Li-salt electrolytes (at the same concentration). The slightly more negative Δ*E*_d_ calculated for [(C_2_F_5_)BF_3_]^−^ compared to [TFSI]^−^ is in good agreement with previous experimental work that showed higher conductivities of IL electrolytes containing [(C_2_F_5_)BF_3_]^−^ compared to [TFSI]^−^ [60,61] and a reported two-fold increase in the ionic conductivity of ethylene carbonate (EC)/ethyl methyl carbonate (EMC) mixtures using Li[(C_2_F_5_)BF_3_] compared to lithium tetrafluoroborate (Li[BF_4_]) [62]. Furthermore, the presence of the nitrile group in [(C_2_F_5_)BF_2_(CN)]^−^ increases both the Δ*E*_d_ max. and Δ*E*_d_ min. values by 35 and 25 kJ/mol, respectively, when compared to [(C_2_F_5_)BF_3_]^−^. This weaker interaction with the Li^+^ supports previous computational findings that showed a decreased interaction energy for boron-based anions with –CN groups as opposed to those with fluorinated functional groups [31]. These more positive Δ*E*_d_ values shown by [(C_2_F_5_)BF_2_(CN)]^−^ compared to [(C_2_F_5_)BF_3_]^−^ may indicate an increase in the ionic conductivity and solubility for the former by facilitating easier Li^+^ dissociation. Therefore, substitution of the –F ligands with –CN groups could provide further improvement in the possible solubility of these heteroleptic anions.

In addition to the enhanced Li^+^ solubility, the weaker Δ*E*_d_ values shown by the borate anions can correspond to an increased participation in SEI layer-forming reactions. This is based on a previous study that proposed that a faster desolvation of the anion from the Li^+^ can lead to an accelerated reaction at the electrolyte and anode interface [63].

**Figure 3:**
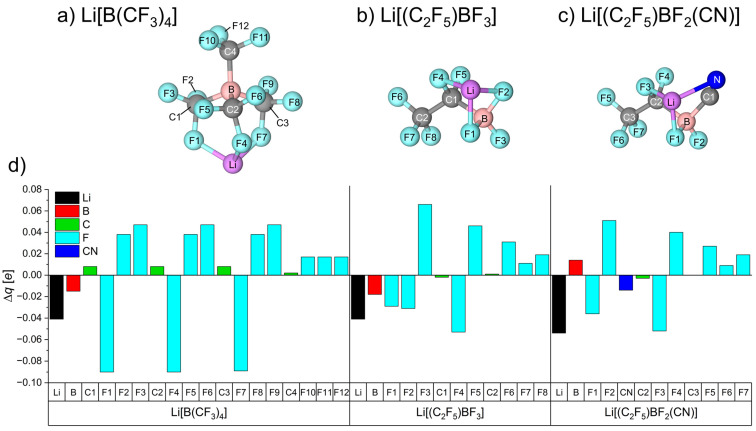
The three most stable Li-salt configurations (**a**–**c**) and the net change in the partial charge (Δ*q*) of the atoms of each Li-salt system (**d**) obtained from natural bond orbital (NBO) analysis.

The geometries of the most stable configurations of the Li-salt systems are shown in Figure 3a–c, with the net change in the partial charge of the atoms (Δ*q*) after coordinating to the Li^+^ shown in Figure 3d.

For Li[B(CF_3_)_4_] in Figure 3a, a single F atom from three separate –CF_3_ moieties is coordinated to the Li^+^ cation. These fluorine atoms (F1, F4 and F7) become more negatively charged, by –0.09*e*, as the Li^+^ withdraws −0.041*e* from the anion (Figure 3d). The remaining F and C atoms of the [B(CF_3_)_4_]^−^ anion all show a net increase in positive charge, demonstrating the charge delocalisation and redistribution that occurs after coordination to the Li^+^.

For Li[(C_2_F_5_)BF_3_] (Figure 3b), the Li^+^ is also coordinated via three F atoms (F1, F2, and F4), resulting in the same amount of charge being donated from the anion to the Li^+^ (−0.041*e*) that was seen for [B(CF_3_)_4_]^−^. Even though both anions coordinate via three F atoms and donate the same amount of charge, a weaker binding energy is obtained for Li[B(CF_3_)_4_] due to the smaller charge of the F atoms of [B(CF_3_)_4_]^−^ (−0.389*e*) compared to the F atoms attached to the B centre of [(C_2_F_5_)BF_3_]^−^ (−0.566 to −0.398*e*) (Table S1). 

For [(C_2_F_5_)BF_2_(CN)]^−^, the substitution of an –F ligand for a less electronegative nitrile group resulted in a less positive charge at the B centre (+0.847*e*) compared to [(C_2_F_5_)BF_3_]^−^ (+1.137*e*) (Figure 3c,d). Upon Li-coordination, only one of the two F atoms (F1) is involved, and the second coordination is afforded to the N atom of the nitrile ligand, while the third coordination is with one of the F atoms of the –C_2_F_5_ group (F3), as occurs for Li[(C_2_F_5_)BF_3_]. For both Li[(C_2_F_5_)BF_2_(CN)] and Li[(C_2_F_5_)BF_3_], Li^+^ coordination leads to a redistribution of charge where the charge of the –F ligand that does not bind with the Li^+^ (atoms F2 and F3, respectively) becomes significantly more positive (Table S1). However, unlike the B atom of Li[(C_2_F_5_)BF_3_], which becomes more negative (Δ*q*(B) = −0.018*e*), the presence of the cyano group in Li[(C_2_F_5_)BF_2_(CN)] caused a small increase in the positive charge of the B atom (Δ*q*(B) = +0.014*e*). This is most likely due to the C1 atom of the CN ligand accepting a charge. We note that the Δ*q*(B) and Δ*q*(C1) values for Li[(C_2_F_5_)BF_2_(CN)] are both equal with 0.014*e*, but with opposite signs. Interestingly, the partial charge of the N atom did not change (Table S1), while the Li^+^ withdrew −0.054*e* from this anion, which is slightly more when compared to the Li[B(CF_3_)_4_] and Li[(C_2_F_5_)BF_3_] systems. However, the weaker interaction energies calculated for [(C_2_F_5_)BF_2_(CN)]^−^ compared to [(C_2_F_5_)BF_3_]^−^ can be attributed to the –CN group being less able to withdraw electrons (Δ*q*(CN) = −0.014*e*) compared to a third electronegative –F ligand found in [(C_2_F_5_)BF_3_]^−^ (Δ*q*(F2) = −0.031*e*). 

**Figure 4:**
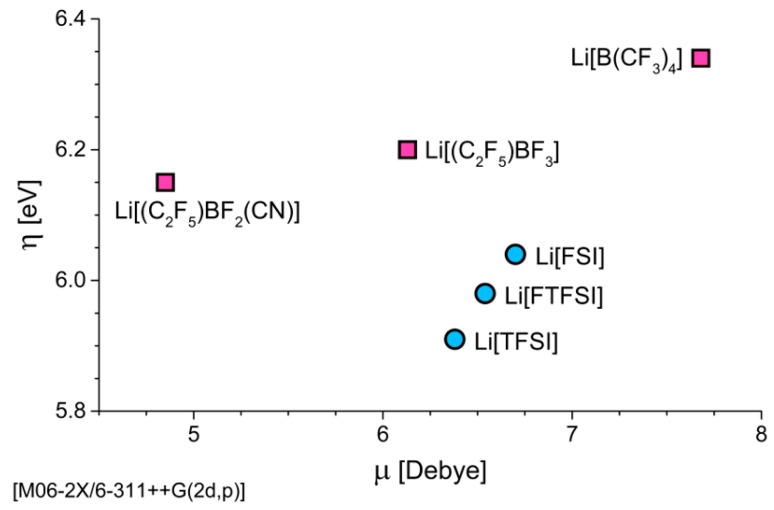
A plot of the chemical hardness (η) versus dipole moment (μ) of the borate anions (squares) and sulfonamide anions (circles).

The smaller dipole moment (μ) of 4.85 D for [(C_2_F_5_)BF_2_(CN)]^−^ (Figure 4) provides further evidence of a weaker interaction with the Li^+^ than for [(C_2_F_5_)BF_3_]^−^ (6.13 D), as a larger dipole moment would correspond to a stronger ion–dipole attraction between the Li^+^ and the anion.

However, we note that a larger dipole moment does not always imply a stronger interaction with the Li^+^. This is shown by the weakly coordinating [B(CF_3_)_4_]^−^ anion having the largest calculated dipole moment (7.68 D) while also possessing the weakest Δ*E*_d_ min. (−524 kJ/mol) of all anions considered in this study. Again, this weaker binding energy of [B(CF_3_)_4_]^−^ is attributed to the reduced charge of the F atoms in the –CF_3_ moieties (−0.398*e*) compared to the more negatively charged F atoms of the –BF_x_ (x = 2 or 3) groups on the individual [(C_2_F_5_)BF_3_]^−^ (−0.566*e*) and [(C_2_F_5_)BF_2_(CN)]^−^ (−0.542*e*) anions. The delocalised negative charge across the four –CF_3_ groups results in the weaker coordination to the Li^+^. 

Overall, all three boron-based anions show delocalisation of the negative charge and, when compared to the sulfonamide anions, have a weaker interaction with the Li^+^, based on the smaller electronic dissociation energy values.

#### 3.1.2. Chemical Hardness

The chemical hardness (η) can be used to indicate the ability of the anion to either donate or accept an electron from the anode. Anions with lower η values would broadly be associated with higher reactivity, which is a desirable characteristic for SEI-forming additives that rely on reduction at the anode [63]. Alternatively, an increased resistance to reduction (larger η values) can correspond to less electrolyte consumption during cycling, which would benefit the longevity of LMBs. Therefore, the chemical hardness can be used as a descriptor to predict the resistance to decomposition reactions occurring at the electrode [64].

The [B(CF_3_)_4_]^−^ anion is shown to have the highest value of chemical hardness (6.34 eV), followed by the [C_2_F_5_)BF_3_]^−^ (6.20 eV) and [(C_2_F_5_)BF_2_(CN)]^−^ (6.15 eV) anions (Figure 4). The lower value of η after the addition of a –CN ligand supports a previous computational study that demonstrated a similar trend for boron-based anions that contain one or more –CN ligands [31]. Compared to the sulfonamide anions, all three borate anions have a greater chemical hardness and therefore would be expected, in a qualitative sense, to be more resistant to decomposition during reduction at the Li anode. However, this does not imply that they will be inert and hence unsuitable for SEI layer formation (see Section 3.2).

#### 3.1.3. Stability against Oxidation and Reduction

**Figure 5:**
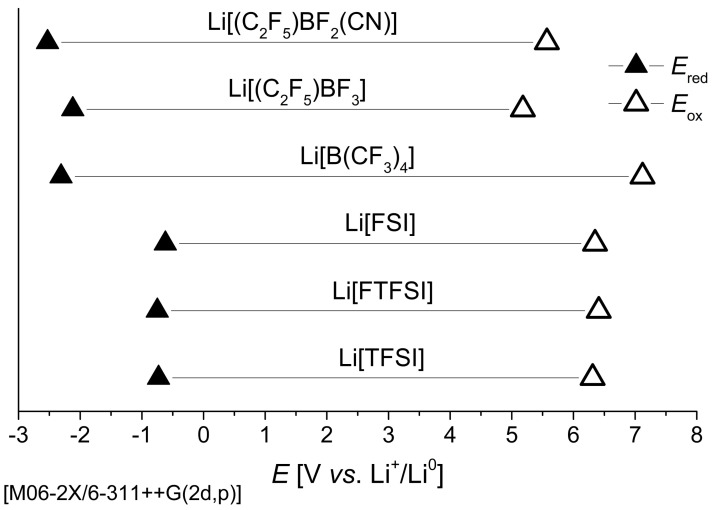
The calculated electrochemical reductive (*E*_red_) and oxidative (*E*_ox_) stability limits of the borate anion-based Li-salts from this work (compared to those based on the sulfonamide anions [FSI]^−^, [FTFSI]^−^, and [TFSI]^−^ from reference [40]).

The calculated oxidative and reductive stability limits of the borate and sulfonamide [40] based Li-salts are shown in Figure 5 and Table S2.

The calculated oxidative stability limit of Li[B(CF_3_)_4_] (7.12 V vs. Li^+^/Li^0^) is significantly higher compared to the heteroleptic borate and sulfonamide anions. The smaller *E*_ox_ values of 5.57 and 5.18 V vs. Li^+^/Li^0^ calculated for Li[(C_2_F_5_)BF_2_(CN)] and Li[(C_2_F_5_)BF_3_] indicate that these anions may be restricted to operating at lower voltages (<5.5 V), such as 4 V class LMBs. In contrast, the calculated *E*_ox_ value of Li[B(CF_3_)_4_] would suggest it is suitable for batteries using high-voltage cathode materials. 

The calculated oxidation potential of Li[(C_2_F_5_)BF_3_] agrees well with the experimental value of 5.3 V vs. Li^+^/Li^0^ for the salt in propylene carbonate on a Pt electrode [62]. To the best of our knowledge, there are currently no experimental oxidative and reductive stability limits reported for the Li[B(CF_3_)_4_] and Li[(C_2_F_5_)BF_2_(CN)] salts in the literature. However, there are previous experimental studies that have shown the *E*_ox_ of the ILs *N*-butyl-*N*-methylpyrrolidinium ([Pyr_14_])[(C_2_F_5_)BF_3_] [34] and 1-ethyl-3-methylimidazolium ([EMIm])[(C_2_F_5_)BF_2_(CN)] [33] to be 2.24 and 3.2 V vs. ferrocene/ferrocenium (Fc^+^/Fc), respectively. Interestingly, the calculations in this study show a similar trend when compared to the experimental *E*_ox_ for [Pyr_14_][(C_2_F_5_)BF_3_] and [EMIm][(C_2_F_5_)BF_2_(CN)] ILs, with the addition of the –CN ligand shown to significantly increase the oxidation stability limit by 0.4 V vs. Li^+^/Li^0^. A similar trend was also observed for the calculated *E*_ox_ of other boron-based anions after the addition of –CN ligands [31]. 

The calculations show all three borate anions to have a significantly more negative reduction potential (<−2.1 V vs. Li^+^/Li^0^) compared to the sulfonamide anions (>−0.8 V vs. Li^+^/Li^0^). The addition of the nitrile group also enhanced the reductive stability limit by −0.4 V vs. Li^+^/Li^0^, resulting in Li[(C_2_F_5_)BF_2_(CN)] being the most stable salt (−2.53 V vs. Li^+^/Li^0^). This estimate agrees well with the experimental value of −2.6 V for the [EMIm][(C_2_F_5_)BF_2_(CN)] IL [33]. It must be noted that the experimental *E*_red_ of [EMIm][(C_2_F_5_)BF_2_(CN)] is limited by the presence of the (relatively) reductively unstable imidazolium cation (rather than the anion), and the reductive stability limit is very likely to be several hundred millivolts more negative in a respective pyrrolidinium salt [17,18,34]. The calculated *E*_red_ of Li[B(CF_3_)_4_] and Li[(C_2_F_5_)BF_3_] were found to be less stable than Li[(C_2_F_5_)BF_2_(CN)], with values of −2.31 and −2.12 V vs. Li^+^/Li^0^, respectively. These values of *E*_red_ calculated for the Li-borate anion pairs are less favourable compared to the sulfonamide anions, indicating the borate anions are more resistant to reduction, which is consistent with their higher calculated chemical hardness.

### 3.2. Li-Salt/Li(001)

To assess the chemical stability and involvement of the borate anions in the formation of an SEI layer, AIMD simulations were performed using the most stable configuration of Li[B(CF_3_)_4_], Li[(C_2_F_5_)BF_3_], and Li[(C_2_F_5_)BF_2_(CN)] adsorbed on the Li(001) surface. These simulations were performed at both 298 and 358 K temperatures to be representative of normal and severe operating conditions, respectively. It is important to mention that these simulations are performed without a negative bias or excess of charge included in the system. Therefore, these systems represent the initial contact of the electrolyte with the Li anode prior to cycling.

#### 3.2.1. Li[B(CF_3_)_4_]/Li(001)

**Figure 6:**
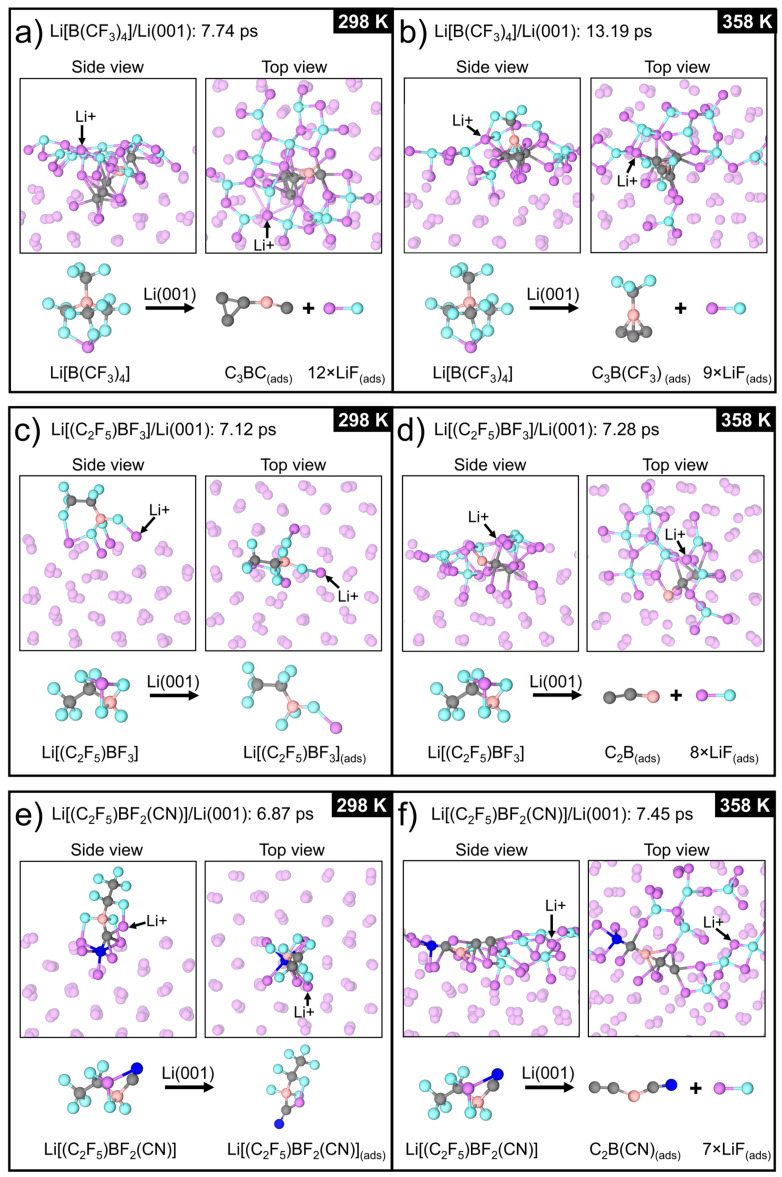
Snapshots of the final structure of the ab initio molecular dynamics (AIMD) simulations of the three different Li-salt/Li(001) systems at room temperature (298 K) (**a**,**c**,**e**) and elevated temperature (358 K) (**b**,**d**,**f**).

The weakly coordinating [B(CF_3_)_4_]^−^ anion broke down at both room and elevated temperatures. The reaction steps of the anion decomposition at the two different temperatures are presented in the supplementary material in Figure S5 (298 K) and Figure S6 (358 K). The final structure and chemical species present at the end of the 298 K and 358 K simulations are shown in Figure 6a,b, respectively.

The [B(CF_3_)_4_]^−^ anion was found to initially adsorb on the Li surface via the F atoms of two of the –CF_3_ groups. This was followed by dissociation of the C–F bond after 0.95 ps (298 K) or 2.01 ps (358 K) from the –CF_3_ groups that were coordinated to the Li surface atoms. The defluorination of these two –CF_3_ groups that were initially coordinated to the Li surface resulted in the spontaneous formation of the heterocyclic compound C_2_B(CF_3_)_2_ after 2.60 ps (298 K) and 3.60 ps (358 K). For the simulation at 298 K, the remaining C–F bonds dissociated successively over the span of ≈1.85 ps, leaving the adsorbed organic compound, C_3_BC, consisting of a three-membered carbocyclic ring (–C_3_) (Figure 6a). In contrast, the simulation at 358 K found an alternative decomposition pathway of the C_2_B(CF_3_)_2_ fragment, leading to C_3_B(CF_3_), which was comprised of a –C_3_B cluster and a single –CF_3_ moiety (Figure 6b). This compound remained adsorbed within the first two layers of the Li surface for the remainder of the simulation, as seen for the C_3_BC compound at 298 K. Based on the simulation at 298 K, cleavage of the remaining C–F bonds of the C_3_B(CF_3_) fragment would likely transpire if the 358 K simulation time were to be extended. Thus, it is expected that the initial SEI layer would be composed predominantly of disordered LiF, with the possible inclusion of carbon–boron-based compounds. As dissociative adsorption occurs readily at room temperature, and given the enhanced calculated electronic properties, [B(CF_3_)_4_]^−^ shows greater potential than the other borate anions in this study as both part of an electrolyte and as an additive for the promotion of in situ SEI layer formation.

#### 3.2.2. Li[(C_2_F_5_)BF_3_]/Li(001)

For the simulation at 298 K, the [(C_2_F_5_)BF_3_]^−^ anion binds via the three F atoms of the –BF_3_ group to two Li surface atoms, as well as the Li^+^, which also adsorbs to the surface, within 0.25 ps. It remains adsorbed and intact for the entirety of the 7.12 ps simulation (Figure 6c). The B–F bonds of [(C_2_F_5_)BF_3_]^−^ are shown to be robust during the simulation, similar to the B–F bonds of [BF_4_]^−^, which previously have been shown to remain intact during the AIMD simulations of Li[BF_4_], [EMIm][BF_4_], and ethylammonium [BF_4_] on the (001) and (100) Li surfaces [43,65,66], even at temperatures > 1500 K [65] (we note that the (001) and (100) facets of Li are equivalent by symmetry). However, the decomposition of [(C_2_F_5_)BF_3_]^−^ does occur at 298 K if the anion is orientated so that the –CF_2_ moiety interacts with the Li surface atoms (Figure S7). Following adsorption of the anion via the F atoms of both the –BF_3_ and –CF_2_ groups, a C–F bond breaks after 3.85 ps. Then, this is followed by dissociation of the C–F bond from the –CF_3_ moiety at 3.91 ps, leaving the parent fragment (CF_2_CF)BF_3_ adsorbed on the surface.

At 358 K, decomposition of the anion was also observed with the reaction mechanism described in Figure S8. The anion adsorbed to the Li(001) surface via two F atoms of the –BF_3_ group, while a F atom from the –CF_2_ and –BF_3_ groups remained coordinated to the adsorbed Li^+^. As shown at 298 K, the simulation at 358 K shows that the interaction of the –CF_2_ group with the Li surface atoms is essential for the anion to break down, with the first C–F bond breaking after 1.79 ps at 358 K. After the initial C–F bond dissociation from the –CF_2_ group, cleavage of a C–F bond from the –CF_3_ moiety then occurred after 1.83 ps. The simultaneous dissociation of a F atom from the –BF_3_ group and the C2 atom occurred after an additional ≈0.5 ps, which was followed by breaking of the remaining C–F bonds at 2.40 and 2.55 ps, leaving a C_2_(BF_2_) fragment embedded in the uppermost three surface layers. The remaining two F atoms eventually dissociated from the B atom after further simulation, resulting in all eight dissociated F atoms contributing to the formation of LiF species on the uppermost layer of the Li(001) surface (Figure 6d). The remaining C_2_B fragment remained intact and embedded in the top three surface layers for the duration of the simulation. 

Thus, the initial SEI layer (prior to cycling) is expected to consist of a disordered LiF layer, which will occur more readily at elevated temperatures.

#### 3.2.3. Li[(C_2_F_5_)BF_2_(CN)]/Li(001)

For the simulation at 298 K, [(C_2_F_5_)BF_2_(CN)]^−^ adsorbed on the Li surface via the two F atoms, and the –CN ligand attached to the B atom, within 0.3 ps. The anion remained intact and tethered onto the Li(001) surface for the entirety of the simulation (Figure 6e). However, similar to [(C_2_F_5_)BF_3_]^−^, C–F bond dissociation was observed at 298 K when the anion adsorbs so that the –CF_2_ moiety interacts with the surface Li atoms (Figure S9). This occurs after 0.42 ps, with the dissociation of a C–F bond from the –CF_2_ group. Then, cleavage of B–F and C–F bonds followed after 0.60 and 0.73 ps of simulation, respectively, leaving a (CF_3_)CBF(CN) fragment adsorbed on the surface. Coordination between the –CF_3_ group with the surface leads to an additional C–F bond cleavage after 0.81 ps of simulation.

Decomposition of the anion also occurred at the elevated temperature, with these reactions described in Figure S10. At 358 K, cleavage of one of the C–F bonds in the –CF_2_ group occurs, identical to [(C_2_F_5_)BF_3_]^−^, with this step occurring after 0.42 or 2.64 ps, depending on the orientation of the adsorbed anion (Figure S11). This first reaction step is followed by the dissociation of B–F and C–F bonds from the B and C3 atoms, all within ≈0.10 ps. The three dissociated F atoms formed LiF surface species, while the parent fragment underwent further decomposition, resulting in the breaking of the remaining C–F and B–F bonds. The final decomposition products formed after 4.59 ps of simulation consist of the seven F atoms that formed LiF species in the two uppermost surface layers and the organo-boron compound C_2_B(CN), which remained embedded in the Li surface (Figure 6f).

Overall, all three borate anions show the ability to provide a LiF-rich SEI layer. Complete defluorination of [B(CF_3_)_4_]^−^ was predicted to occur at 298 K and 358 K. All F atoms from the [(C_2_F_5_)BF_3_]^−^ and [(C_2_F_5_)BF_2_(CN)]^−^ anions formed LiF species only during simulations at 358 K; however, it is expected that longer simulations at 298 K would have also captured their entire defluorination. After the anions had adsorbed on the surface, the first decomposition step of all three borate anions involved the cleavage of a C–F bond. The C–F bonds of the borate anions all break at approximately the same time (<2 ps). For the sulfonamide anions [TFSI]^−^ and [FSI]^−^ that have been previously simulated on the Li surface (Table 1) [44,65,67], the simulation time preceding their initial decomposition reactions is approximately an order of magnitude faster when compared to the initial decomposition step of the borate anions. This could be because the IL pair was optimised prior to performing the AIMD simulations [65,67].

Based on the AIMD simulations, the chemical stability of the borate anions, when in contact with the clean Li anode, is expected to be similar to that of the [TFSI]^−^ and [FSI]^−^ anions. However, the borate anions are likely to have superior reductive stability, as indicated by the calculated chemical hardness and the reductive stability limits. Therefore, unwanted continuous decomposition at the passivated anode surface is less likely to occur during cycling of the borate anions. Additionally, SEI layer formation reactions for these borate anions are unlikely to form undesirable gaseous products such as SO_2_ that can originate from [TFSI]^−^. Avoiding the generation of gaseous products from side reactions between the electrolyte and the Li anode is essential for maintaining the integrity and uniformity of the SEI layer, as has been amply demonstrated in the study by Shkrob et al. [68] for [FSI]^−^. Hence, the fact that only adsorbed borocarbons and LiF species are predicted to form during the decomposition reactions at the Li anode is a promising feature of the borate anions studied in this work. Only the simulations of Li[B(CF_3_)_4_]/Li(001) produced boron-based compounds with homo- and heterocyclic rings, with these species notably absent from the Li[(C_2_F_5_)BF_3_]/Li(001) and Li[(C_2_F_5_)BF_2_(CN)]/Li(001) systems. The boron-based compounds formed after the decomposition of all three anions were embedded in the Li surface, which could indicate that a B-rich SEI layer is possible for all three anions at room and elevated temperatures. The formation of both a F- and a B-rich SEI layer using these anions may help stabilise the Li anode during cycling, as has been shown to occur for lithium difluoro(oxalate)borate (Li[DFOB]) [26]. Furthermore, nitrile-containing electrolytes, such as succinonitrile, have been associated with stabilising high-voltage cathode materials through the formation of a protective cathode electrolyte interphase layer via their decomposition at the cathode surface [69]. Therefore, the breakdown products of the nitrile-containing [(C_2_F_5_)BF_2_(CN)]^−^ anion could help stabilise the surface of high-voltage cathode materials, improving the cycling stability of LMBs.

## 4. Conclusions

We have shown that the borate anions [B(CF_3_)_4_]^−^, [(C_2_F_5_)BF_3_]^−^, and [(C_2_F_5_)BF_2_(CN)]^−^ have an advantageous interaction with the Li^+^ cation and the Li anode surface, revealing these anions as a promising addition to the electrolyte components of LMBs. All three anions exhibit a weaker interaction with the Li^+^ cation when compared to the sulfonamide anions, which could result in faster desolvation of the Li^+^ that leads to improved ionic conductivity and solubility. The borate anions are all shown to preferentially form a tri-dentate coordination with the Li^+^ via the F atoms of [B(CF_3_)_4_]^−^ and [(C_2_F_5_)BF_3_]^−^ and the two F atoms and the N atom of [(C_2_F_5_)BF_2_(CN)]^−^. The high reductive stability limits and chemical hardness of the borate anions means they are less likely to undergo reductive decomposition at the Li anode compared to Li[TFSI], Li[FTFSI], and Li[TFSI], which corresponds to less electrolyte consumption during cycling. The calculated oxidative stability limit of all Li-salts is >5 V vs. Li^+^/Li^0^; however, Li[B(CF_3_)_4_] exceeds even Li[FTFSI] with 7.12 V vs. Li^+^/Li^0^. Hence, Li[B(CF_3_)_4_] is an especially promising candidate for high-voltage LMB applications.

The initial decomposition step of the borate anions involved the cleavage of a C–F bond followed by further defluorination of the anions and the formation of an LiF-rich SEI layer. The breakdown of the anions also resulted in no gaseous by-products and instead adsorbed boron-based compounds: C_3_BC, C_3_(BCF_3_), C_2_B, or C_2_B(CN). These findings show the borate anions to have an advantageous reaction with the Li anode and can stabilise the anode surface by forming an in situ LiF-rich SEI layer. Including these borate anions in electrolytes as part of a Li-salt or IL by pairing with the most stable [Pyr_14_]^+^, phosphonium ([R_4_P]^+^), or boronium ([NNBH_2_]^+^) cations provides a new strategy for the development of practical LMBs due to the superior electrochemical stability, ionic conductivity, and potential Li anode protection that the anions can facilitate.

## Figures and Tables

**Table 1:** The temperature and time when key bonds break in the borate and sulfonamide anions during AIMD simulations on the Li(001) surface.

System [cation][anion]	Temp. (K)	Time (ps)	Bond Broken	Reference
Li[B(CF_3_)_4_]	298	0.95	C–F	This study
	358	2.01	C–F	
Li[(C_2_F_5_)BF_3_]	298	3.85	C–F	This study
	358	1.79	C–F	
		2.35	B–F	
Li[(C_2_F_5_)BF_2_(CN)]	298	0.42	C–F	This study
		0.60	B–F	
	358	0.37	C–F	
		2.70	B–F	
[NNBH_2_][TFSI]	298	0.50	C–F	[67]
	358	2.25	N–S	
[Pyr_14_][TFSI]	298	∼0.08	S–C	[65]
			N–S	
[Pyr_13_][FSI]	298	0.05	S–F	[44]
Sulfolane-Li[BF_4_]	300	65	–	[66]
	450	65	–	
[EMIm][BF_4_]	289	35	–	[65]
	2500	N/A	B–F	

## Data Availability

The data presented in this study are available on request from the corresponding author. The data are not publicly available due to technical and time limitations.

## Supplementary Materials

The following are available online at https://www.mdpi.com/article/10.3390/nano11092391/s1, Figure S1: The optimised [B(CF_3_)_4_]^−^ anion, the 26 Li^+^ binding sites sampled, and the two minimum energy configurations of Li[B(CF_3_)_4_], Figure S2: The optimised [(C_2_F_5_)BF_3_]^−^ anion, the 21 Li^+^ binding sites sampled, and the six minimum energy configurations of Li[(C_2_F_5_)BF_3_], Figure S3: The optimised *anti*-[(C_2_F_5_)BF_2_(CN)]^–^ anion with the 21 Li^+^ binding sites sampled and the six minimum energy configurations of Li[(C_2_F_5_)BF_2_(CN)], Figure S4: The optimised *syn*-[(C_2_F_5_)BF_2_(CN)]^–^ anion with the 25 Li^+^ binding sites sampled and the eight minimum energy configurations of Li[(C_2_F_5_)BF_2_(CN)]^−^, Figure S5: The decomposition pathway of the [B(CF_3_)_4_]^–^ anion at 298 K, Figure S6: The decomposition pathway of the [B(CF_3_)_4_]^−^ anion at 358 K, Figure S7: The decomposition pathway of the [(C_2_F_5_)BF_3_]^−^ anion at 298 K, Figure S8: The decomposition pathway of the [(C_2_F_5_)BF_3_]^−^ anion at 358 K, Figure S9: The decomposition pathway of the [(C_2_F_5_)BF_2_(CN)]^−^ anion at 298 K, Figure S10: The decomposition pathway of the [(C_2_F_5_)BF_2_(CN)]^−^ anion at 358 K, Figure S11: The initial decomposition step of [(C_2_F_5_)BF_2_(CN)]^−^ as determined during the AIMD simulations at 358 K, Table S1: Calculated partial charges of the Li^+^ and atoms of the borate anions, Table S2: Structural and electronic properties of the three borate anion based Li-salts.
