# Supplementary material for: Fluorinated Boron-Based Anions for Higher Voltage Li Metal Battery Electrolytes
Source: Nanomaterials (Basel). 2021 Sep 14;11(9):2391. doi: 10.3390/nano11092391 (PMC8466916; doi:10.3390/nano11092391)
Supplement: Supplementary file 1 [file nanomaterials-11-02391-s001.zip › nanomaterials-1373745-supplementary.pdf]

Supplementary Material

Fluorinated Boron-Based Anions for Higher Voltage Li Metal Battery Electrolytes

Jonathan Clarke-Hannaford [1], Michael Breedon [2,*], Thomas Rüther [3] and Michelle J.S. Spencer [1,4,*]

[1] School of Science, RMIT University, GPO Box 2476, Melbourne, Victoria 3001, Australia; jonathan.clarke-hannaford@student.rmit.edu.au
[2] CSIRO Manufacturing, Private Bag 10, Clayton South, Victoria 3169, Australia
[3] CSIRO Energy, Private Bag 10, Clayton South, Victoria 3169, Australia; thomas.ruether@csiro.au
[4] ARC Centre of Excellence in Future Low-Energy Electronics Technologies (FLEET), School of Science, RMIT University, GPO Box 2476, Melbourne, Victoria 3001, Australia
* Correspondence: michael.breedon@csiro.au (M.B.); michelle.spencer@rmit.edu.au (M.J.S.S.)

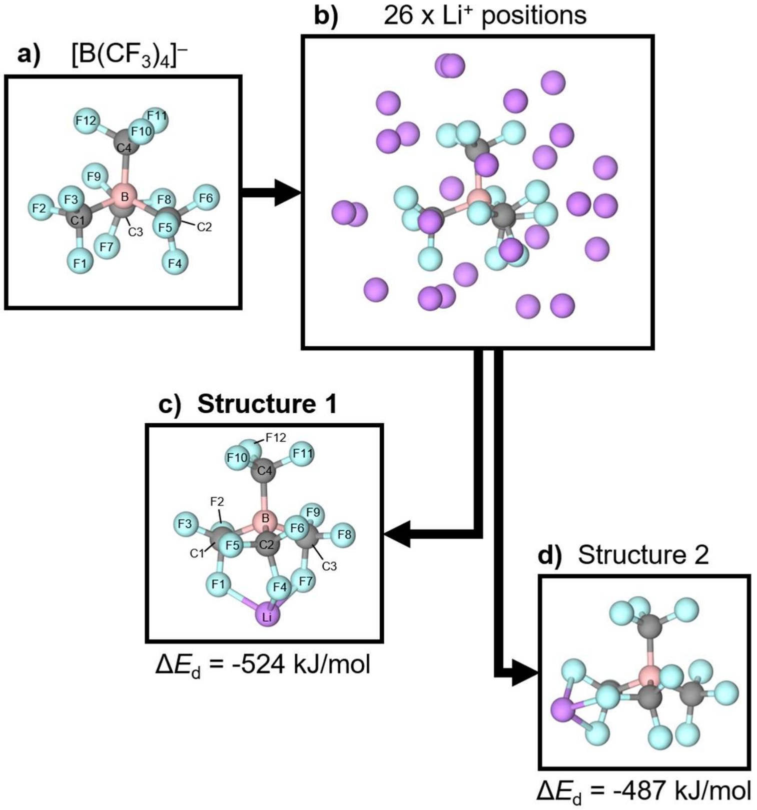

Figure S1. The (**a**) optimised $[B(CF_3)_4]^-$ anion, the (**b**) 26 Li^+ binding sites sampled, and the two minimum energy configurations (**c–d**) of $Li[B(CF_3)_4]$.

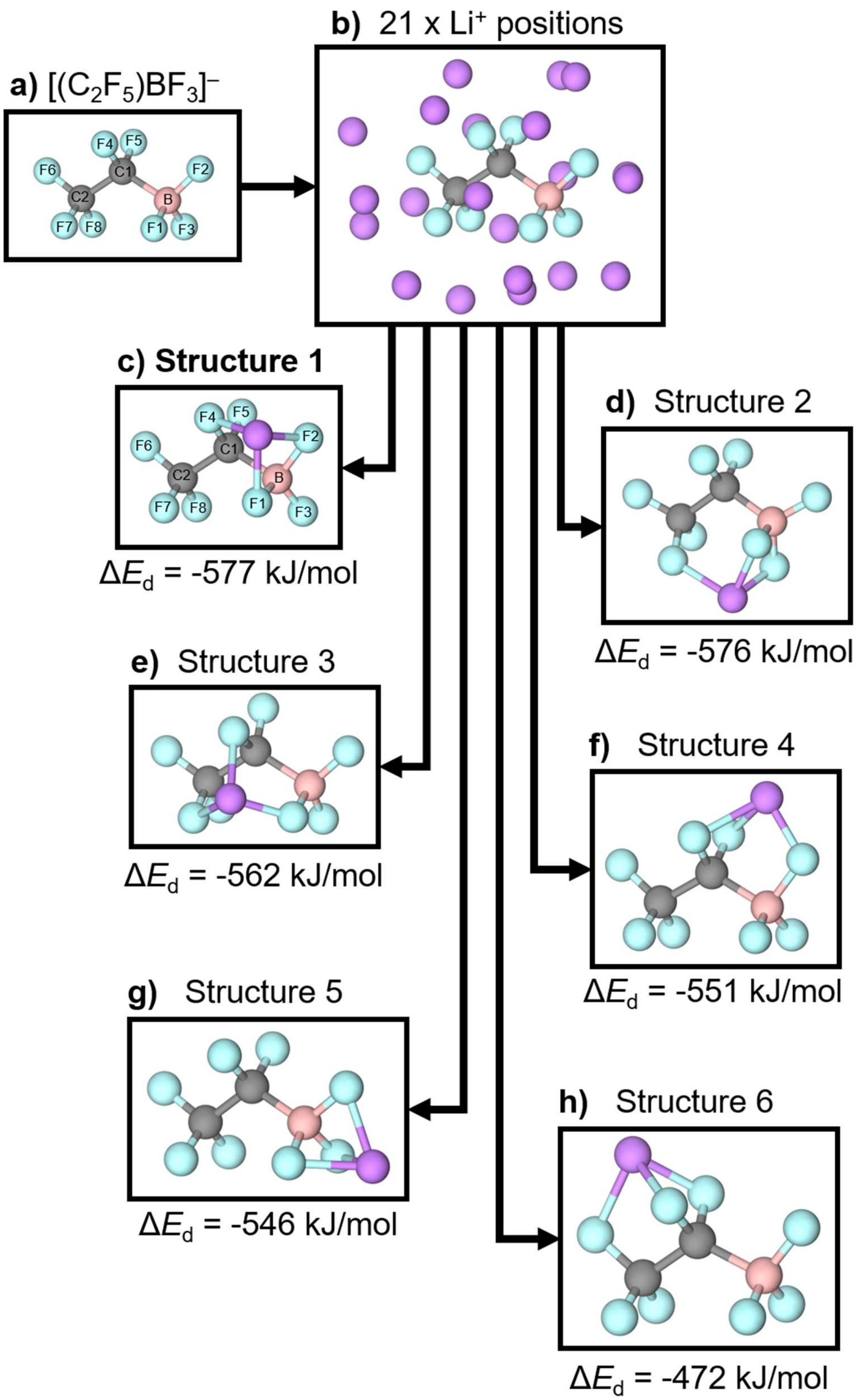

Figure S2. The (**a**) optimised $[(C_2F_5)BF_3]^-$ anion, the (**b**) 21 Li^+ binding sites sampled, and the six minimum energy configurations (**c-h**) of $Li[(C_2F_5)BF_3]$.

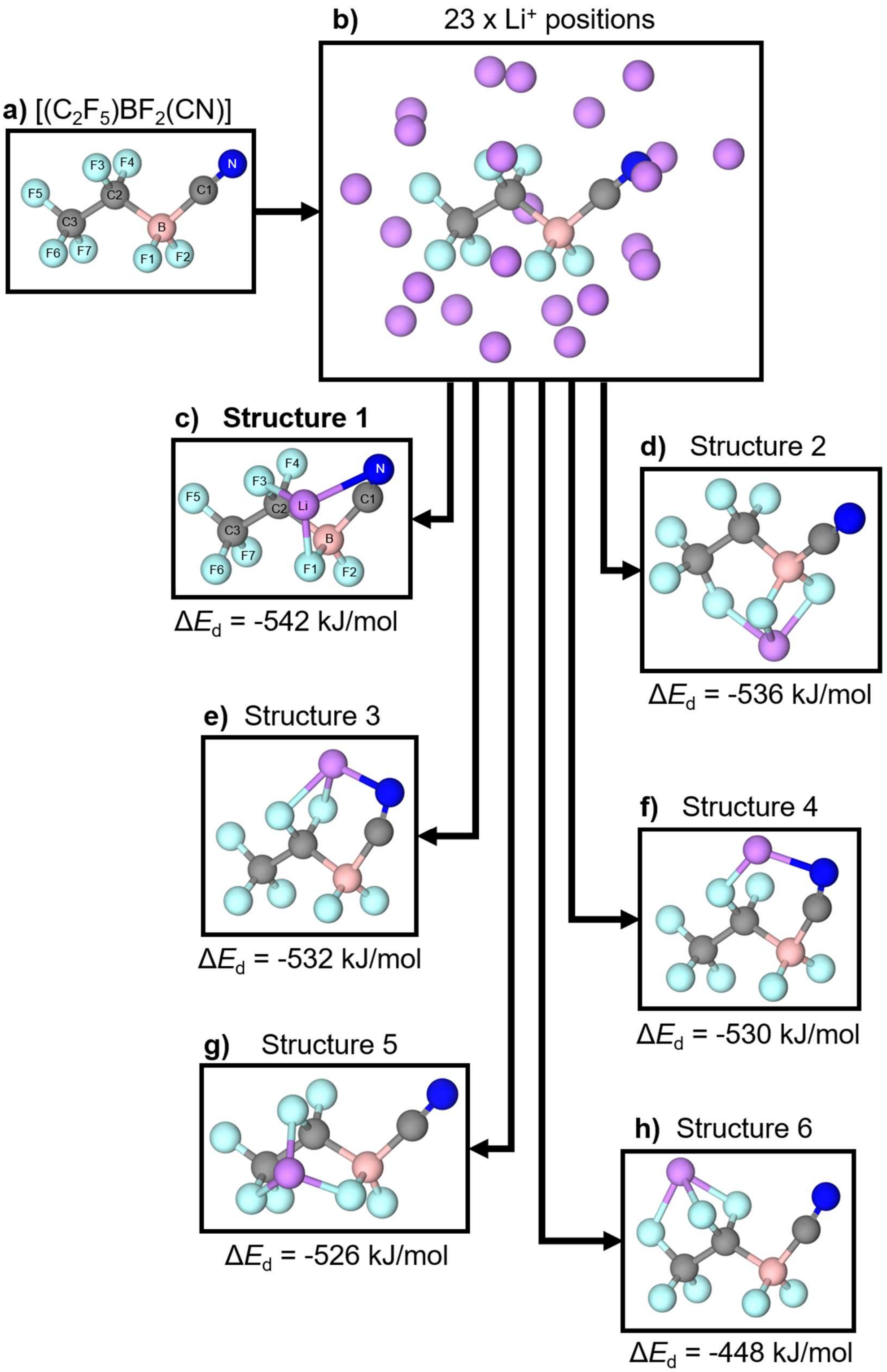

Figure S3. The (**a**) optimised *anti*-[$(C_2F_5)BF_2(CN)]^-$ anion with the (**b**) 21 Li^+ binding sites sampled, and the six minimum energy configurations (**c-h**) of $Li[(C_2F_5)BF_2(CN)]$.

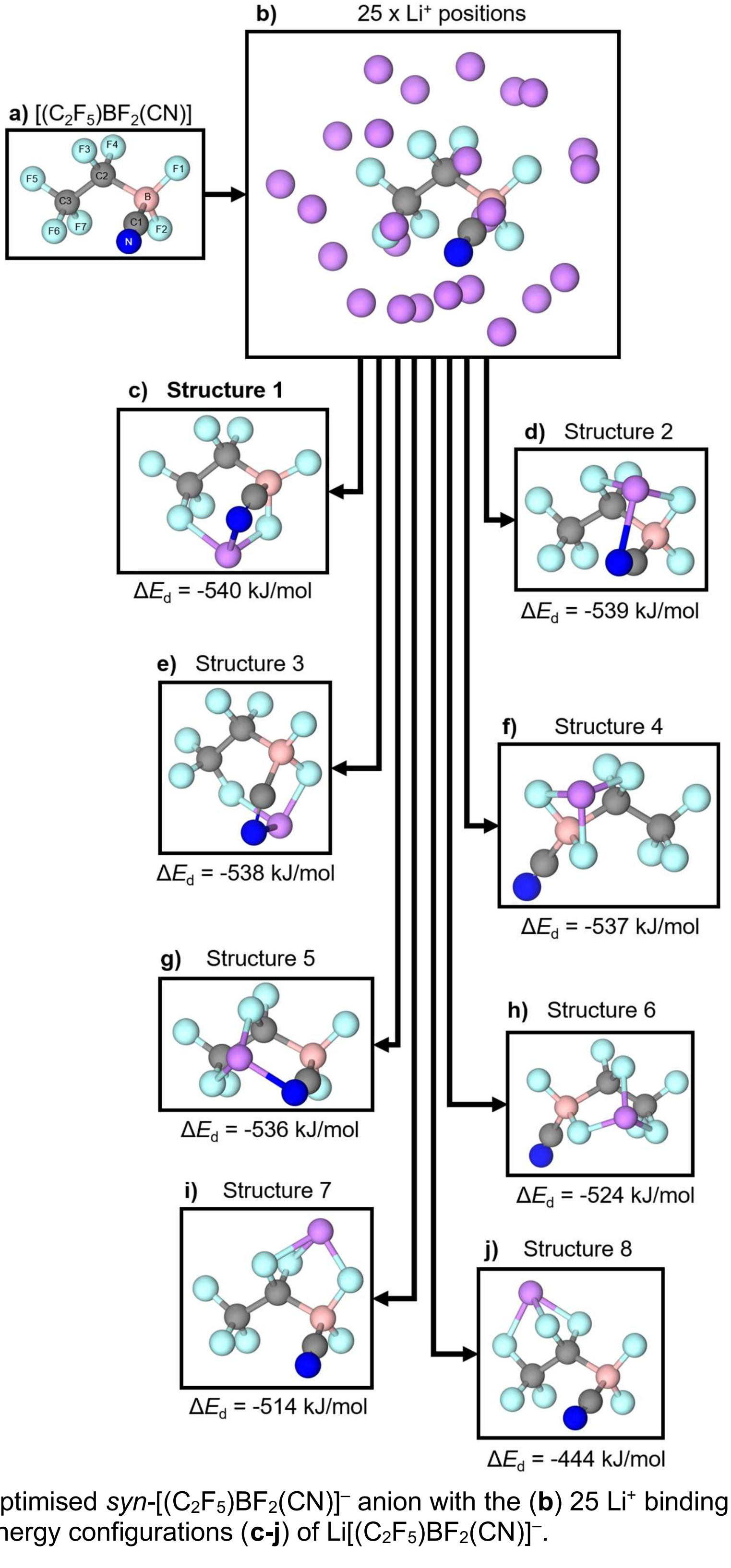

Figure S4. The (**a**) optimised *syn*-$[(C_2F_5)BF_2(CN)]^-$ anion with the (**b**) 25 Li^+ binding sites sampled, and the eight minimum energy configurations (**c-j**) of $Li[(C_2F_5)BF_2(CN)]^-$.

Table S1. Calculated partial charges of the Li^+ and atoms of the borate anions when separate (Li^+ + $[anion]^-$), and of the most stable Li-salt system ($Li[anion]^-$). The net change in charge (Δq) that occurs for the individual atoms after the Li^+ binds to the anion is also shown. All values are shown in electrons, *e*.

System	Li	B	N	C1	C2	C3	C4	F1	F2	F3	F4	F5	F6	F7	F8	F9	F10	F11	F12
$Li[B(CF_3)_4]$																			
Li^+ + $[anion]^-$	+1.000	-0.095	–	+0.940	+0.940	+0.940	+0.940	-0.389	-0.389	-0.389	-0.389	-0.389	-0.389	-0.389	-0.389	-0.389	-0.389	-0.389	-0.389
Li[anion]	+0.959	-0.110	–	+0.948	+0.948	+0.948	+0.942	-0.479	-0.350	-0.342	-0.479	-0.350	-0.342	-0.478	-0.351	-0.342	-0.371	-0.371	-0.371
Δq	-0.041	-0.015	–	+0.008	+0.008	+0.008	+0.002	-0.090	+0.038	+0.047	-0.090	+0.038	+0.047	-0.089	+0.038	+0.047	+0.017	+0.017	+0.017
$Li[(C_2F_5)BF_3]$																			
Li^+ + $[anion]^-$	+1.000	+1.137	–	+0.427	+1.044	–	–	-0.566	-0.564	-0.566	-0.398	-0.398	-0.385	-0.365	-0.365	–	–	–	–
Li[anion]	+0.959	+1.119	–	+0.425	+1.045	–	–	-0.595	-0.595	-0.501	-0.452	-0.353	-0.354	-0.354	-0.346	–	–	–	–
Δq	-0.041	-0.018	–	-0.002	+0.001	–	–	-0.029	-0.031	+0.066	-0.053	+0.046	+0.031	+0.011	+0.019	–	–	–	–
$Li[(C_2F_5)BF_2CN]$ (*anti*)																			
Li^+ + $[anion]^-$	+1.000	+0.847	-0.447	+0.066	+0.452	+1.051	–	-0.542	-0.542	-0.391	-0.391	-0.379	-0.362	-0.362	–	–	–	–	–
Li[anion]	+0.946	+0.861	-0.447	+0.053	+0.449	+1.050	–	-0.578	-0.491	-0.443	-0.352	-0.352	-0.353	-0.343	–	–	–	–	–
Δq	-0.054	+0.014	0.000	-0.014	-0.003	0.000	–	-0.036	+0.051	-0.052	+0.040	+0.027	+0.009	+0.019	–	–	–	–	–

Table S2. Structural and electronic properties of the three borate anion based Li-salts studied in this work and sulfonamide based Li-salts from ref. 1.

Li[Anion]	$d_{(Li\text{-}A)}$ [Å]	ΔE_d min. [kJ mol^{-1}]	ΔE_d max. [kJ mol^{-1}]	μ [Debye]	IP_a [eV]	EA_a [eV]	IP_v [eV]	EA_v [eV]	χ [eV]	η [eV]	E_{ox} [V *vs* Li^+/Li^0]	E_{red} [V *vs* Li^+/Li^0]	ESW [V]
This work:													
$Li[B(CF_3)_4]$	1.84	-524	-487	7.68	10.96	-0.60	12.27	-0.41	5.93	6.34	7.12	-2.31	9.43
$Li[(C_2F_5)BF_3]$	1.86	-577	-473	6.13	9.49	-0.51	12.09	-0.30	5.90	6.20	5.18	-2.12	7.30
$Li[(C_2F_5)BF_2(CN)]$	1.88	-542	-448	4.85	9.40	-0.61	12.03	-0.26	5.89	6.15	5.57	-2.53	8.10
From ref. 1													
Li[TFSI]	1.79	-590	-493	6.38	10.20	-0.46	11.48	-0.35	5.57	5.91	6.31	-0.73	7.04
Li[FSI]	1.80	-587	-501	6.70	10.63	-0.47	11.74	-0.34	5.70	6.04	6.35	-0.62	6.97
Li[FTFSI]	1.79	-588	-497	6.54	10.51	-0.46	11.60	-0.35	5.63	5.98	6.41	-0.75	7.16

Shortest lithium-anion distance ($d_{(Li\text{-}A)}$). The minimum (ΔE_d min.) and maximum (ΔE_d max) electronic dissociation range. Dipole moment (μ). The adiabatic ionisation potential (IP_a) and electron affinity (EA_a). The vertical ionisation potential (IP_v) and electron affinity (EA_v). Electronegativity (χ). Chemical hardness (η). The calculated oxidative stability limit (E_{ox}) and reductive stability limit (E_{red}). The calculated electrochemical stability limit (ESW).

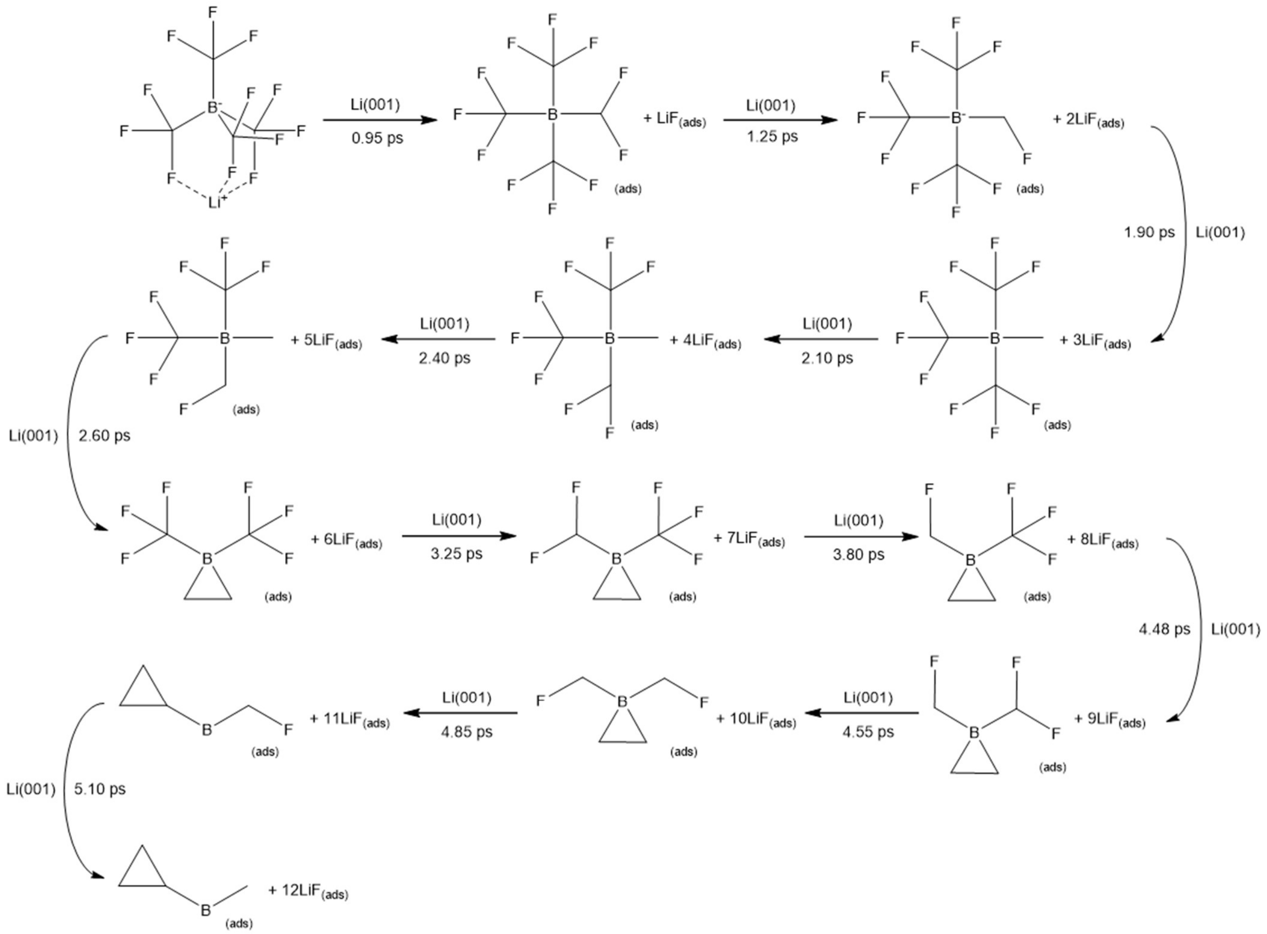

Figure S5. The decomposition pathway of the $[B(CF_3)_4]^-$ anion (as determined during the AIMD simulations) at 298 K.

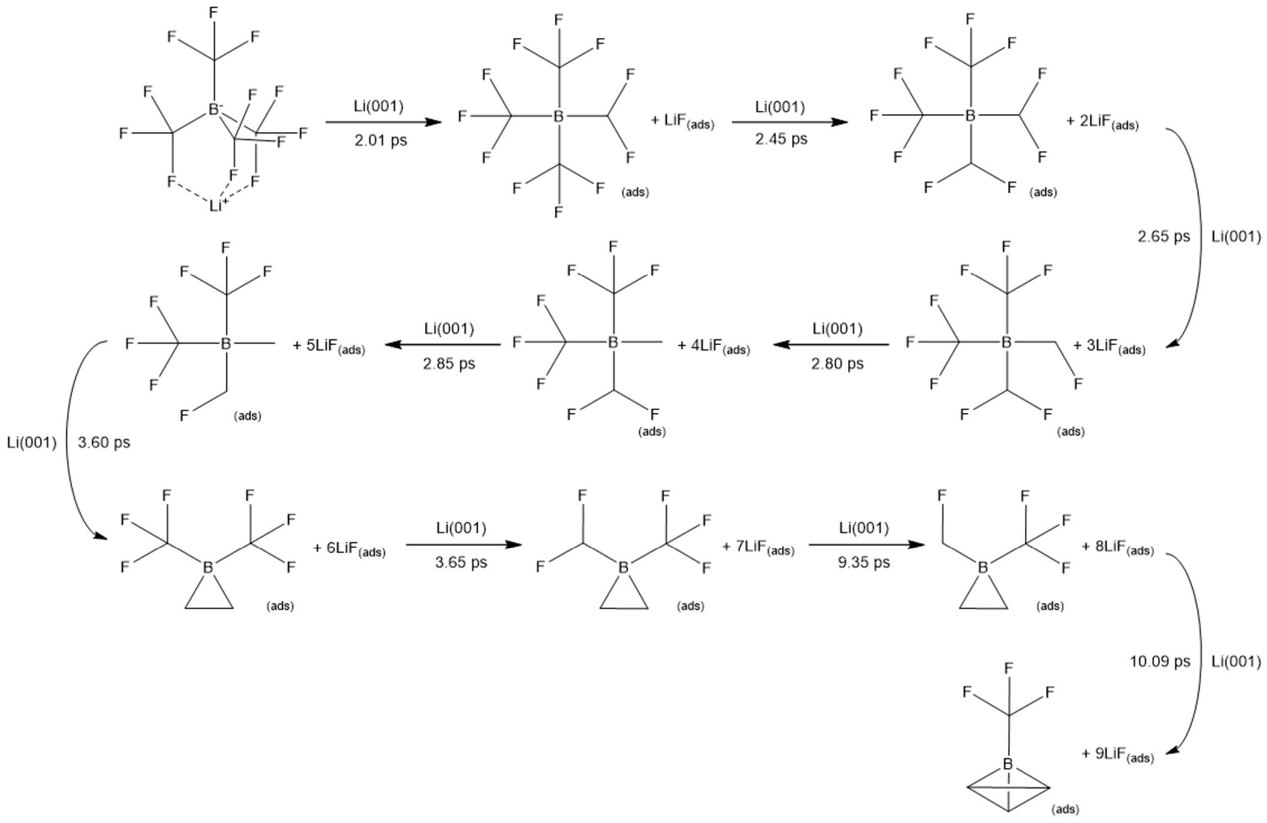

Figure S6. The decomposition pathway of the $[B(CF_3)_4]^-$ anion (as determined during the AIMD simulations) at 358 K.

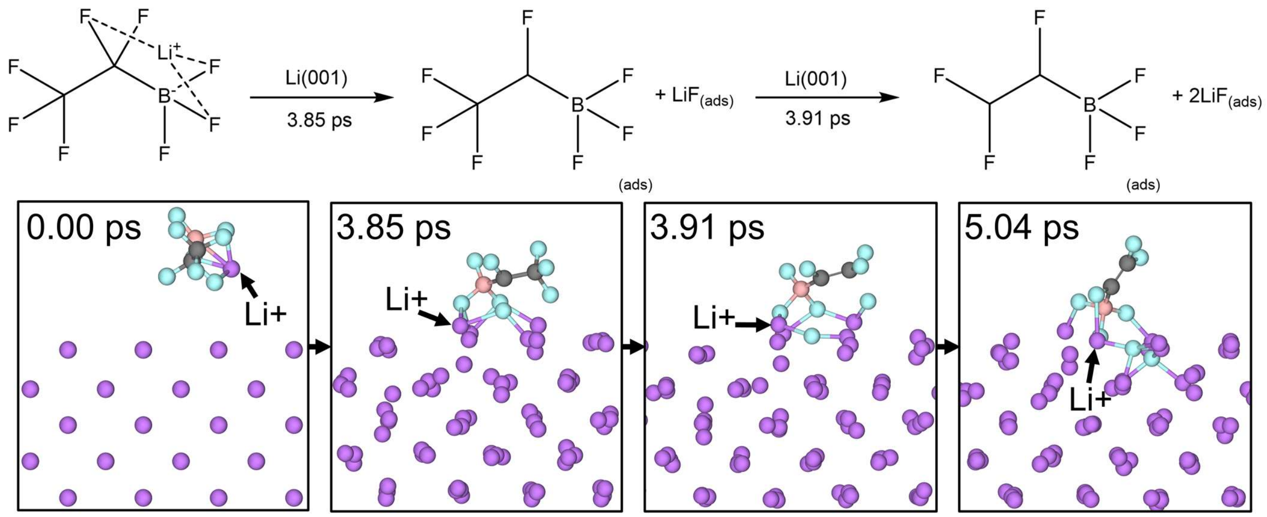

Figure S7. The decomposition pathway of the $[(C_2F_5)BF_3]^-$ anion (as determined during the AIMD simulations) at 298 K.

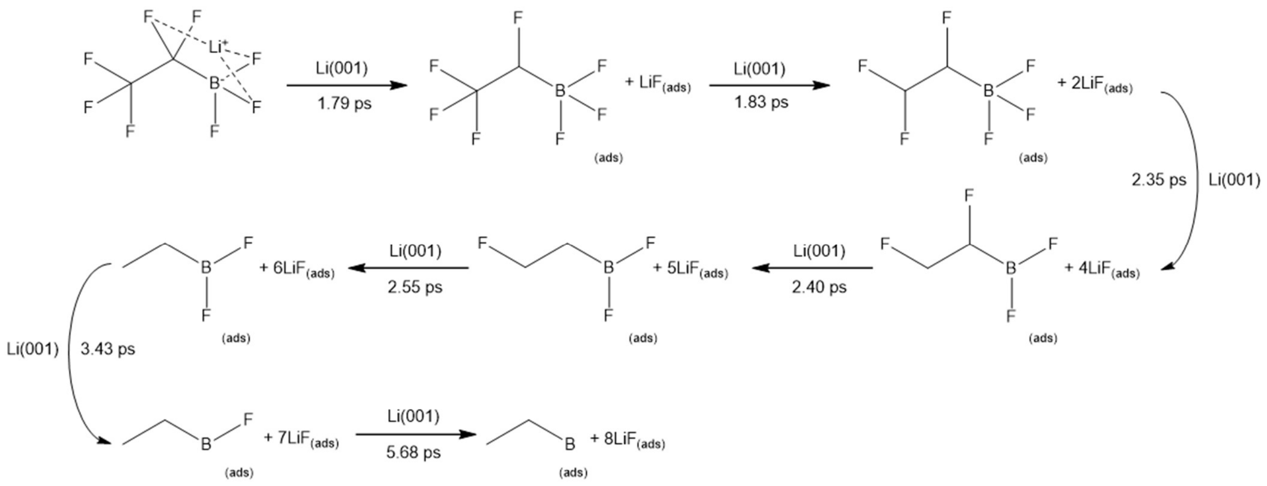

Figure S8. The decomposition pathway of the $[(C_2F_5)BF_3]^-$ anion (as determined during the AIMD simulations) at 358 K.

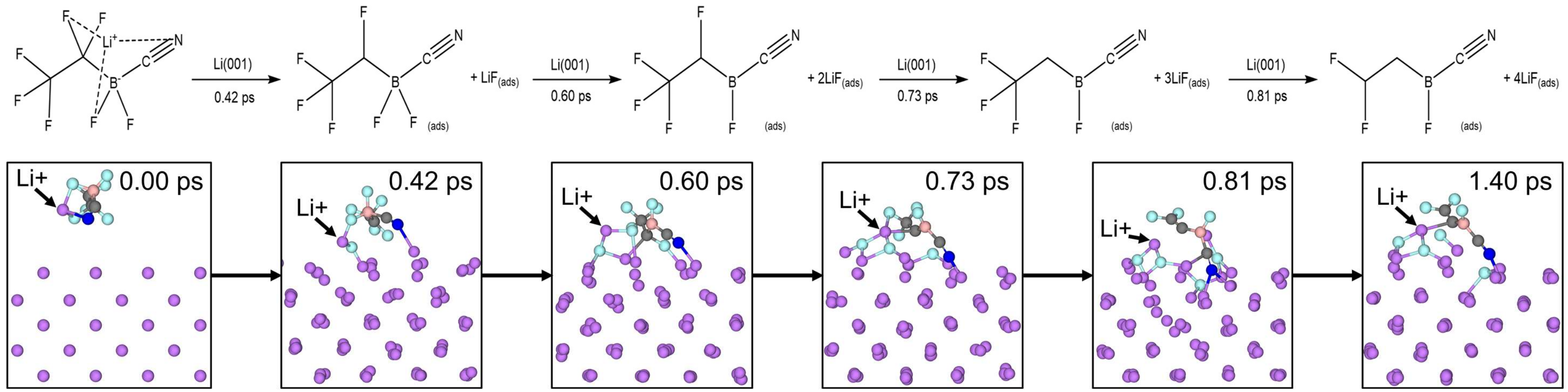

Figure S9. The decomposition pathway of the $[(C_2F_5)BF_2(CN)]^-$ anion (as determined during the AIMD simulations) at 298 K.

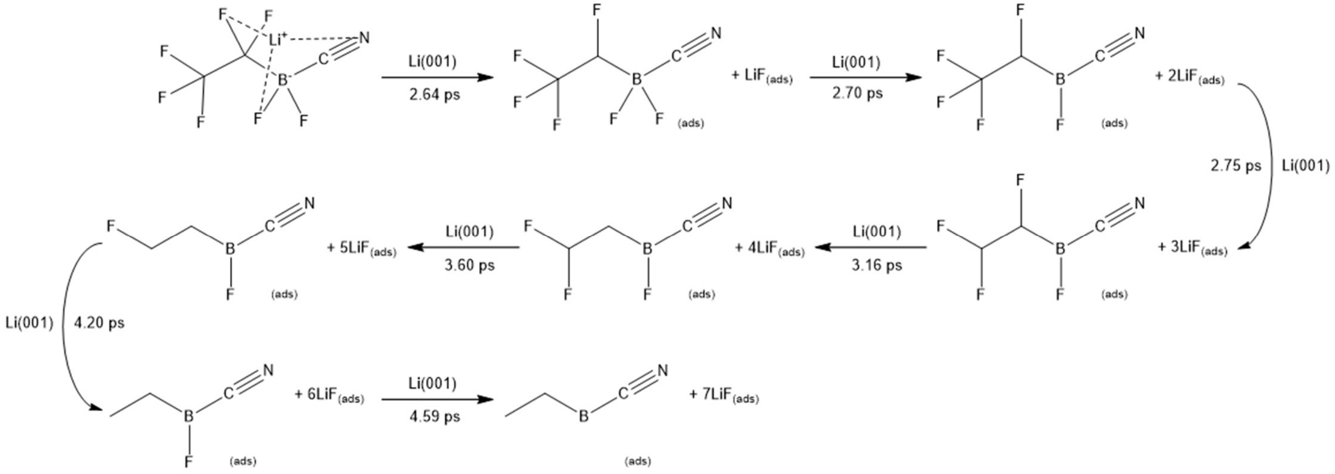

Figure S10. The decomposition pathway of the $[(C_2F_5)BF_2(CN)]^-$ anion (as determined during the AIMD simulations) at 358 K.

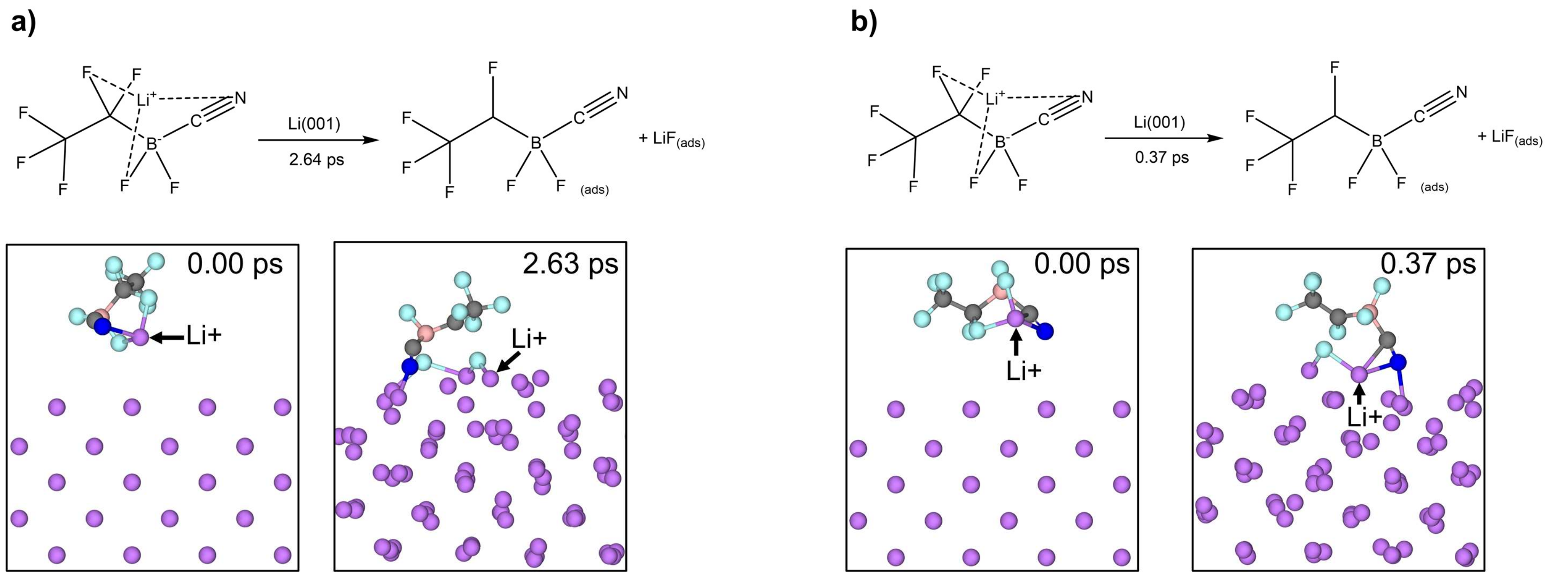

Figure S11. The initial decomposition step of $[(C_2F_5)BF_2(CN)]^-$ as determined during the AIMD simulations at 358 K when the $–CF_2$ moiety is orientated (**a**) away from or (**b**) towards the Li(001) surface.

REFERENCES

[1] D. A. Osborne, M. Breedon, T. Rüther, M. J. S. Spencer, submitted for publication in J. Phys. Chem. C, 2021.
